# Discrimination of *Gardnerella* Species by Combining MALDI-TOF Protein Profile, Chaperonin *cpn60* Sequences, and Phenotypic Characteristics

**DOI:** 10.3390/pathogens10030277

**Published:** 2021-03-01

**Authors:** Aistė Bulavaitė, Thomas Maier, Milda Pleckaityte

**Affiliations:** 1 Institute of Biotechnology, Life Sciences Center, Vilnius University, Sauletekio al. 7, 10257 Vilnius, Lithuania; aiste.bulavaite@bti.vu.lt; 2 R&D Bioanalytics, MALDI Biotyper Business Area Microbiology & Diagnostics, Bruker Daltonik GmbH, Fahrenheitstr. 4, 28359 Bremen, Germany; thomas.maier@bruker.com

**Keywords:** *Gardnerella*, bacterial vaginosis, species, *cpn60* sequences, MALDI-TOF, sialidase, phenotypic characteristics

## Abstract

The description of *Gardnerella vaginalis* was recently updated and three new species, including nine genome species within *Gardnerella*, were defined using whole genome sequences and matrix assisted laser desorption ionization time of flight (MALDI-TOF) mass spectrometry. A fast and simple method based on readily available techniques would be of immense use to identify *Gardnerella* species in research and clinical practice. Here we show that 34 previously characterized *Gardnerella* isolates were assigned to the species using partial chaperonin *cpn60* sequences. The MALDI Biotyper from Bruker Daltonik GmbH demonstrated the capability to differentiate the phylogenetically diverse groups composed of *G. vaginalis*/*G. piotii* and *G. leopoldii*/*G. swidsinskii*. Among the phenotypic properties that characterize *Gardnerella* species are sialidase and β-galactosidase activities. Our data confirmed that the NanH3 enzyme is responsible for sialidase activity in *Gardnerella* spp. isolates. Almost all *G. piotii* isolates displayed a sialidase positive phenotype, whereas the majority of *G. vaginalis* strains were sialidase negative. *G. leopoldii* and *G. swidskinskii* displayed a sialidase negative phenotype. β-galactosidase is produced exclusively in *G*. *vaginalis* strains. Earlier determined phenotypic characteristics associated with virulence of *Gardnerella* isolates now assigned to the defined species may provide insights on how diverse species contribute to shaping the vaginal microbiome.

## 1. Introduction

*Gardnerella vaginalis* has been the only identified species in the genus *Gardnerella* for a long time. While this bacterium is found to be closely associated with bacterial vaginosis (BV), a form of vaginal dysbiosis [1,2], *Gardnerella* isolates from BV-positive women showed genetic and phenotypic diversity [3,4,5]. *Gardnerella* has also been detected in vaginal microbial communities of healthy BV-negative women [1,6]. These findings suggest a diverse role of genetic variants of *Gardnerella* in the vaginal microbiota.

The earlier proposed biotyping [7] and genotyping [8] schemes had limited success revealing *Gardnerella* diversity. The comparative genomic analysis of the 17 genomes allowed separating *Gardnerella* isolates into 4 subgroups, which likely are separate species [9,10]. The existence of four subgroups within *Gardnerella* was confirmed by the sequence analysis of *cpn60* gene [11,12]. In 2019, Vaneechoutte and colleagues [13] performed the genome analysis (digital DNA–DNA hybridization (DDH) and average nucleotide identity (ANI)) of 81 whole genomes of *Gardnerella* isolates and proposed the genus separation into four species: *Gardnerella vaginalis*, *Gardnerella piotii*, *Gardnerella leopoldii*, *Gardnerella swidsinskii*, and 9 genome species. This work also confirmed earlier findings that *Gardnerella* spp. cannot be differentiated based on the 16S rRNA gene sequences as they share no less than 98.5% sequence similarity. While all *G. vaginalis* isolates corresponded to previously described subgroup 1, subgroup 2 included *G. piotii* and genome species 3, species *G. leopoldii* and *G. swidsinskii* corresponded to subgroup 4, and subgroup 3 contained at least three neither named nor formally described species most probably due to the low number of isolates [13]. The colonies of four named *Gardnerella* species had the same appearance on blood agar plates, but the differences in β-galactosidase and sialidase activities were determined.

Selection of a fast, simple, and not expensive method based on readily available techniques would be of great use to identify *Gardnerella* species in research and clinical practice. In this study, we aimed to differentiate 34 previously characterized *Gardnerella* isolates of known subgroups/clades [14,15,16] into newly defined species and genome groups [13] using matrix-assisted laser desorption/ionization time-of-flight (MALDI-TOF) mass spectrometry and chaperonin *cpn60* universal target (UT) sequences [11]. We also determined how the presence of the genes coding for sialidases NanH1, NanH2, and NanH3 reflects the ability of *Gardnerella* species to display sialidase activity. Assigning the previously determined phenotypic features [16] of three subgroups/clades to the newly defined *Gardnerella* species provide an understanding of how these species may impact the development of vaginal dysbiosis.

## 2. Results and Discussion

### 2.1. Collection of Gardnerella Isolates

Thirty-three *Gardnerella* isolates from the characterized vaginal samples were subtyped previously [14] based on the subgroup/clade-specific genes as described earlier [9,10]. The strain GV37 was isolated from blood [15] and its whole genome sequence was deposited in GenBank (acc. no. CP019058.1). *Gardnerella* isolates were assigned to three subgroups (clade 1, clade 2, and clade 4), whereas isolate 86.1 was negative in all clade-specific PCR assays and defined as an unknown subgroup [14]. The phenotypic characteristics of the isolates and their distribution among subgroups were determined previously: the in vitro ability to produce the toxin vaginolysin, to form a biofilm and express sialidase activity [16]. Vaginolysin was quantified using a monoclonal antibody-based sandwich ELISA. The amount of biofilm produced in brain-heart infusion broth with supplements (BHIs) in 96-well microplate was quantified by safranin staining. The presence of the sialidase A gene was determined by PCR, whereas the sialidase activity in culture supernatants of *Gardnerella* isolates was quantified using fluorogenic substrate [16].

The resolving power of protein profiling by MALDI-TOF and partial chaperonin *cpn60* sequences were used for the separation of 34 *Gardnerella* isolates into the newly defined species [13].

### 2.2. Differentiations of Gardnerella Species Based on cpn60 UT Sequences

**Figure 1:**
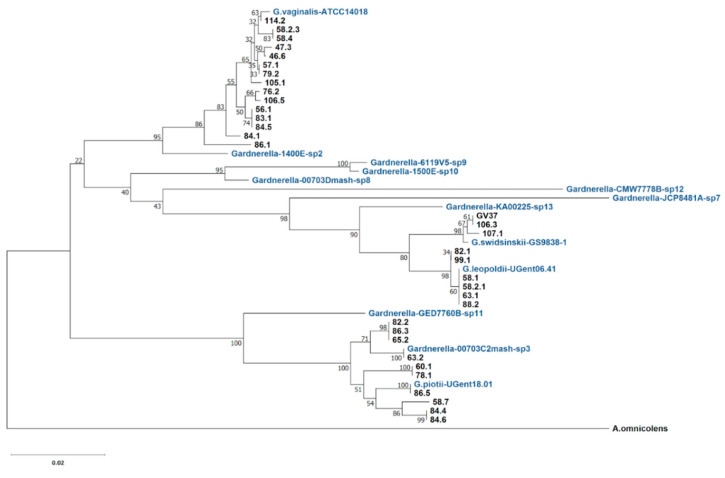
Phylogenetic relationships of 34 *Gardnerella* spp. isolates based on *cpn60* UT sequences. The type strains of *G. vaginalis*, *G. piotii*, *G. swidsinskii*, *G. leopoldii* species and nine genome species [13] were included. Evolutionary history was inferred using the neighbor-joining method [18]. The percentage of the replicate trees in which the associated taxa clustered together in the bootstrap test of 500 replicates is indicated. *Alloscardovia omnicolens* sequence was included as an outgroup [17]. Evolutionary analyses were conducted in MEGA X [19].

It was demonstrated that chaperonin *cpn60* universal target sequences of 552 bp are a perfect tool for determining *Gardnerella* subgroups [12] and the newly defined species and genome species [17]. In this study, *Gardnerella* isolates were differentiated in the phylogenetic tree based on *cpn60* UT sequences (Figure 1). The reference sequences from the type strains of four named species and nine genome species [13] were included. Fifteen isolates that correspond to the previously determined subgroup/clade 1 and *G. vaginalis* type strain (ATCC 14018) share the branch in the tree. Although *G. vaginalis* and genome species 2 share the same node, they are well separated with high bootstrap support. Ten isolates were clustered with *G. piotii* type strain. Four strains (63.2, 65.2, 82.2, and 86.3) from this cluster and genome species 3 type strain share the same node. The separation of subgroup/clade 2 isolates into *G. piotii* and genome species 3 was consistent with the phylogenetic relationship described earlier [17].

The isolates corresponding to the previously determined subgroup/clade 4 were separated into *G. swidsinskii* and *G. leopoldii* species by MALDI-TOF and whole genome comparison [13]. Six isolates (58.1, 58.2.1, 63.1, 82.1, 88.2, and 99.1) were grouped with *G. leopoldii* type strain (UGent 06.41) and three (106.3, 107.1, and GV37) with *G. swidsinskii* type strain (GS9838-1)*,* although both species displayed close relationship in the phylogeny that is in agreement with the results described in [17]. The GV37 isolate with known whole genome sequence has previously been attributed to *G. swidsinskii* [13]. Any other genome species except for genome species 3 and four named species were not identified among the isolates.

Pairwise distances between the *cpn60* UT nucleotide sequences were calculated (Table S1). In contrast to the data obtained by Hill et al. [17], we found strains with identical *cpn60* UT sequence (Table S1). The sequence of isolate 63.2 fully matched the sequence of genome species 3 type strain 00703C2mash-sp3. Isolates 58.1, 58.2.1, 63.1, 88.2, and *G. leopoldii* type strain (UGent 06.41) had identical sequence. Isolate 86.5 and *G. piotii* type strain share the same *cpn60* UT. The following pairs and triplets of isolates had identical *cpn60* UTs: 58.2.3 and 58.4; 57.1 and 79.2; 56.1, 83.1 and 84.5; GV37 and 106.3; 82.1 and 99.1; 82.2, 86.3 and 65.2; 60.1 and 78.1; 84.4 and 84.6. The isolates sharing the same partial *cpn60* sequences represent different strains that were verified by a random amplified polymorphic DNA (RAPD) analysis performed previously [16].

### 2.3. Resolution of Gardnerella Species Based on MALDI Biotyper Protein Profiling

Vaneechoutte and colleagues [13] demonstrated that *Gardnerella* species could be distinguished by MALDI-TOF mass spectrometry, an indispensable tool for clinical microbiology laboratories. Four *Gardnerella* species can be separated mainly into pairs of *G. vaginalis*/*G. piotii* and *G. leopoldii*/*G. swidsinskii* based on their MALDI spectra. Table 2 in the paper by Vaneechoutte et al. 2019 [13] showed seven peak variations (1 single peak and 3 peak pairs) which differentiate *G. vaginalis* and *G. piotii* species: two peak pairs (at mass-to-charge (*m*/*z*) 4422/4429 and 8842/8857 representing single and double-charged ions of the same masses) and the presence/absence of the single peak at *m*/*z* 5162, and the peak pair at *m*/*z* 6855/6885. A unique peak at *m*/*z* 2704 was proposed to be characteristic to resolve *G. leopoldii* and *G. swidsinskii* species. The presence/absence of a single peak at *m*/*z* 5349 and the peak pairs at *m*/*z* 4849/4928/(9795/9853) differentiate between *G. vaginalis*/*G. piotii* and *G. leopoldii*/*G. swidsinskii* [13].

**Figure 2:**
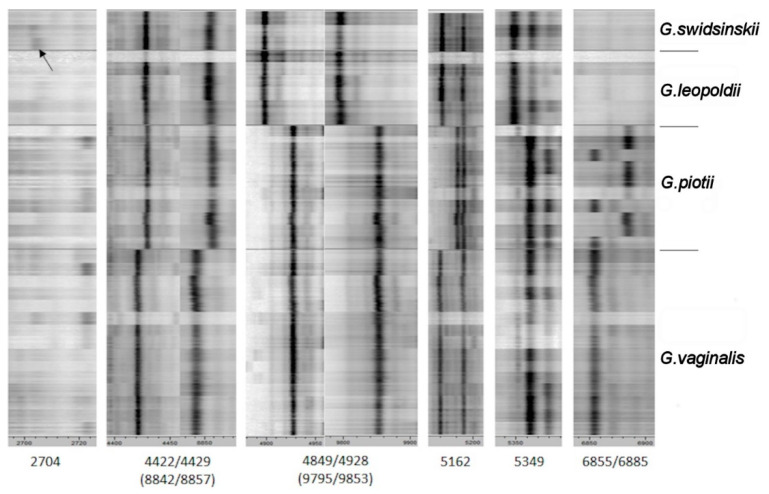
Mass spectra (*n*~600) of 34 *Gardnerella* strains. Peaks at mass-to-charge (*m*/*z*) were indicated according to [13]. The arrow indicates the peak at *m*/*z* 2704.

Protein profiling of 34 *Gardnerella* isolates was performed using the MALDI Biotyper (Bruker Daltonik GmbH) mass spectrometer. The recorded 34 MALDI spectra sets were named based on their *cpn60* UT sequences and grouped together. Fifteen *G. vaginalis*, 10 *G. piotii*/genome species 3, 6 *G. leopoldii* and 3 *G. swidsinskii* strains were subjected for analysis. Figure 2 shows the mass peaks at *m*/*z* in the mass spectrum of *Gardnerella* strains.

**Figure 3:**
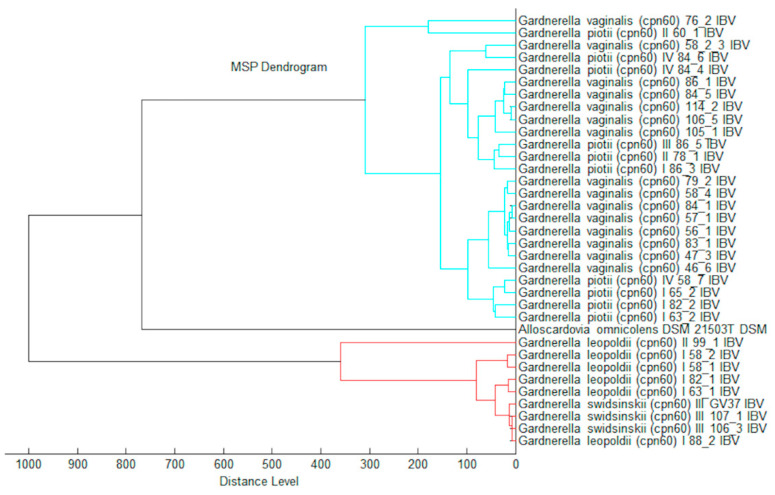
Matrix assisted laser desorption ionization (MALDI) Biotyper log(score) based dendrogram.

All reference spectra (Main Spectrum Profile, MSP) were used for calculation of log(scores) (Table S2) against each other as a taxonomical distance. The log(score) distance was used to demonstrate the taxonomical relationship of *Gardnerella* strains based on a MALDI Biotyper dendrogram (Figure 3).

In this study, the peak at *m*/*z* 2704 proposed as a unique mark to differentiate *G. leopoldii* and *G. swidsinskii* species [13] was observed as a very faint peak (indicated by the arrow in Figure 2). Any other peaks suitable for separation of these two species were not determined. Further, the log(score) based routine identification (Table S2) showed the same result.

The single peak at *m*/*z* 5162 (Figure 2) is a specific mark for *G. vaginalis* and *G. piotii* differentiation. Further, the peak pairs at *m*/*z* 4422/4429/(8842/8857) could be used as species-specific signals. The peak pair at *m*/*z* 6855/6885 did not increase the discriminatory power as several strains of *G. piotii* had a peak at 6855 that was characteristic of *G. vaginalis* in the previous study [13]. In general, very close taxonomic relation of *G. vaginalis* and *G. piotii* was demonstrated by the MALDI Biotyper log(score) algorithm for routine species differentiation (Table S2).

Any characteristic peaks suitable to resolve *G. piotii* and genome species 3 were not observed.

The group *G. leopoldii*/*G. swidsinskii* can be distinguished reliably from the group *G. vaginalis*/*G. piotii* based on the log(scores) routine identification using the MALDI Biotyper. In the future, *G. vaginalis* could be separated from *G. piotii* via the creation of an automated subtyping/differentiation module. Currently, the manual peak picking and peak comparing to the published data could be alternatively used for species discrimination. The next library updates for automated MALDI Biotyper identification will include two species: *Gardnerella vaginalis* containing the matching hint closely related to *Gardnerella piotii* and the species *Gardnerella leopoldii*/*Gardnerella swidsinskii*.

Thus, the *cpn60*-based approach showed the capability to separate *G. vaginalis*, *G. piotii*/genome species 3, *G. swidsinskii*, and *G. leopoldii* species (Figure 1). However, the dendrogram of the MALDI-TOF MS profiles generated using the MALDI Biotyper differentiated the phylogenetically diverse groups composed of species of *G. vaginalis*/*G. piotii* and *G. leopoldii*/*G. swidsinskii* (Figure 3). The fact that *G. vaginalis* and *G. piotii* were not distinguished implies the close relatedness of these species in the genus at the proteome level.

### 2.4. Phenotypic Characteristics of Gardnerella Species

Sialidase activity is an important phenotypic characteristic of *Gardnerella* spp. connected with mucus degradation and the development of BV clinical features [20,21,22]. Although the gene *nanH1* coding for sialidase NanH1 (former sialidase A [23]) was found in sialidase activity positive strains of *Gardnerella* subgroups/clades 1, 2, and 3, the gene was also detected in activity-negative strains raising the question about alternative genes responsible for activity or regulation of the *nanH1* expression [12,16]. Recently two additional sialidases NanH2 and NanH3 with a broad range of activity were detected in *Gardnerella* spp. [23]. The genes *nanH2*, *nanH3* or both were found in activity-positive strains, but absent in activity-negative isolates. All this suggests that these enzymes, but not NanH1, are the primary sources of sialidase activity [23]. Sialidase activity in *Gardnerella* spp. was found to be cell-associated or secreted [20]. Protein organization predicts that NanH2 is a secreted enzyme, NanH3 may be intracellular and/or secreted, whereas NanH1 most probably is intracellular [23].

We found that all *G. swidsinskii*/*G. leopoldii* strains did not contain the *nanH1-nanH2-nanH3* genes and they were sialidase activity-negative (Figure S1; Table S3) by both quantitative filter spot (this study) and the qualitative fluorometric [16] assays. Even though the *nanH1* gene was found in all *G. vaginalis* strains (*n* = 15), the sialidase-positive isolates (*n* = 3) encoded NanH3, except for strain 58.2.3, which was activity negative although possessed *nanH3.*

None of *G. vaginalis* isolates contained the *nanH2* gene (Figure S1). Nine of ten *G. piotii*/genome species 3 strains exhibited sialidase activity. The *nanH3* gene was found in all sialidase activity-positive isolates except for *G. piotii* 86.5, which contained *nanH1* and *nanH2*. Isolate 60.1 was activity-negative, although contained the *nanH3* gene (Figure S1).

Among sialidase-positive strains of *Gardnerella* species, we did not find strains containing solely *nanH1.* The *nanH2* gene was most often found together with *nanH3*. Our data confirm the recent findings [23], that the sialidase-positive phenotype correlates with the presence of *nanH3*. The gene coding for NanH3 prevails in *G. piotii* and the closely related genome species 3, but it is less common in *G. vaginalis*. We agree with the assumption that *G. vaginalis* could gain *nanH3* from *G. piotii* through horizontal gene transfer (HGT) [24]. *G. vaginalis* participates more frequently in HGT [24] acquiring the genes from other *Gardnerella* species that co-exist in vaginal microbiota [10,14,17].

The characteristic feature of *G. vaginalis* is a β-galactosidase activity that is consistent with the data obtained by Vaneechoutte and colleagues [13]. This activity was not found to be present in neither *G. piotii*/genome species 3 nor *G. leopoldii*/*G. swidsinskii* strains.

The phenotypic characteristics previously performed in vitro of three *Gardnerella* subgroups/clades [16] were assigned to the newly differentiated species (*G. vaginalis*, *G. piotii*/*genome species 3*, and *G. swidsinskii*/*G. leopoldii*) (Table S3). *G. vaginalis* strains except 86.1 contained the *vly* gene and produced toxin vaginolysin as well as expressed the ability to form a biofilm, but a minority (3/15) of strains was sialidase-positive. A vast majority of *G. piotii*/genome species 3 strains produced a sialidase and developed a biofilm, whereas the *vly* gene was absent from nearly half the strains. It was proposed that vaginolysin is not a part of a core genome and may be lost or gained by *Gardnerella* species [24]. The characteristic feature of *G. leopoldii*/*G. swidsinkii* strains is a sialidase activity-negative phenotype. However, the specification of additional isolates is required to differentiate closely related *G. leopoldii* and *G. swidsinkii* species.

The *Gardnerella* isolates classified into species were previously isolated from vaginal samples of BV-positive and BV-negative women [14]. We updated the table provided in [16] placing the species name for each isolate (Table S4). The vast majority of vaginal samples contained multiple *Gardnerella* clades, however, we isolated the strains of single or several species from these samples. Overall, five *Gardnerella* species were found in vaginal samples. The strains of three species (*G. vaginalis*, *G. piotii*, and *G. leopoldii*) isolated from the vaginal sample 058S1 (Nugent score = 9) matched the clades identified in that sample by PCR (Table S4). However, some clades (e.g., clade 2) contain several species [13]. A low abundance of particular species in vaginal samples and cultivation issues may result in a loss of isolates. The recent identification of *Gardnerella* species in noncultured vaginal samples based on the *cpn60* UT sequences [17] showed that the most frequently detected species are four named species and genome species 3, an observation that is in agreement with our data. Genome species 2 and 7 to 13 were rarely detected in vaginal samples [17].

## 3. Materials and Methods

### 3.1. Bacterial Strains and Cultivation Conditions

*Gardnerella* spp. isolates were obtained from characterized vaginal samples of women from Lithuania [14]. Bacterial stocks were stored at –80 °C in tryptic soy broth (TSB) (Liofilchem, Roseto degli Abruzzi, Italy) supplemented with 20% (*v*/*v*) horse serum (Oxoid, Thermo Fisher Scientific, Waltham, MA, USA) and 15% (*v*/*v*) glycerol. The isolates were revived on chocolate agar with Vitox (Oxoid) and incubated at 37 °C in 6% CO_2_ and 15% O_2_ atmosphere (CO_2_ Gen, Oxoid) for 48 h. The isolates 58.2.3, 58.4, 84.4, 84.6, 86.1, and 78.1 were incubated for 48 h in anaerobic conditions generated by AnaeroGen (Oxoid).

### 3.2. Sequencing of cpn60 Universal Target Regions

The bacterial suspension in water was repeatedly frozen and thawed. After centrifugation, the supernatant was used for PCR. Enzymes and kits were obtained from Thermo Fisher Scientific (Vilnius, Lithuania). The amplification of *cpn60* sequence was carried out with primers H729 and H730 [11] using Maxima Hot Start Taq DNA polymerase or Dream Taq Hot Start Taq polymerase in the reaction volume of 15 µL. The reactions included denaturation at 94 °C for 4 min, 40 amplification cycles consisting of denaturation for 30 s at 95 °C, annealing for 30 s at 48 °C, and extension for 30 s at 72 °C. The final extension step was prolonged for 2 min. The PCR products were purified using the GeneJET PCR Purification Kit and sequenced with primer Seq-H729 (5′-CGCCAGGGTTTTCCCAGTCACGAC) to identify the 552-bp universal target (UT) sequence of the *cpn60* gene [25]. The *cpn60* UT sequences were deposited at GenBank (accession numbers MT501265–MT501298).

### 3.3. Phylogenetic Analysis

*cpn60* UT sequences from the type strains of four named *Gardnerella* species (*G. vaginalis, G. piotii, G. leopoldii,* and *G. swidsinskii*) and nine genome species [13] were obtained from Chaperonin Database Search (cpnDB) (http://www.cpndb.ca/search.php)(accessed 13 October 2020). A phylogenetic tree based on 552-bp *cpn60* UT was built and visualized using MEGA X [19]. Pairwise distances between the sequences were calculated by MEGA X. The type strain *Alloscardovia omnicolens* (DSM 21503) was included as a root [17].

### 3.4. MALDI-TOF MS—MALDI Biotyper

For MALDI-TOF MS (Matrix One Assisted Laser Desorption/Ionization Time of Flight Mass Spectrometry) analysis, 1 µL inoculation loop of fresh bacterial cells was suspended in 75% ethanol and stored at –20 °C until further processing. The cell suspensions in ethanol were centrifuged at 13.000× *g* for 2 min, the supernatant was discarded and the residual sample centrifuged again for a short time. The remaining ethanol was discarded and the cell pellet was carefully suspended in 50 µL of 70% formic acid followed by the addition of 50 µL acetonitrile. After mixing, the suspension was centrifuged at 13.000× *g* for 2 min. One µL of supernatant was transferred to the disposable MALDI target plate (MSP Biotarget 96, Bruker Daltonik GmbH, Bremen, Germany). Eight replicates of each sample were loaded on the plate, dried at room temperature, and overlaid with 1 µL HCCA (a-cyano-4-hydroxycinnamic acid) matrix solution (Bruker Daltonik GmbH). Each assay included the Bruker Bacterial Test Standard (BTS). Spectra for each sample on the target plate were acquired three times, thus resulting in 24 individual MALDI spectra for each strain. All MALDI measurements were performed using the Bruker standard measurement procedures (standard flexControl method, standard AutoX method, standard MBT-Process method) without any alterations. After spectra quality check (QC) and internal recalibration the MALDI Biotyper standard algorithms were used to create the reference spectra (MSPs).

### 3.5. Detection of the nanH2 and nanH3 Genes and Sialidase Activity by a Filter Spot Test

The *nanH2* and *nanH3* genes were detected by PCR using primers and cycling conditions described in [23]. A qualitative filter paper spot test using cultures in duplicate was applied as described previously [26].

### 3.6. β-galactosidase Activity

A colorimetric assay with o-nitrophenol-beta-d-galactosidase (ONPG) tablets (Sigma Aldrich) according to the manufacturer’s instructions was used to detect β-galactosidase activity of *Gardnerella* spp. strains.

## 4. Conclusions

The recent amendment of the *Gardnerella* taxonomic description prompted us to deploy tools for differentiation of characterized 34 *Gardnerella* isolates of known clade/subgroup into species. Here several techniques were used for species discrimination. Four named *Gardnerella* species and genome species 3 were resolved in the phylogenetic tree based on *cpn60* UT sequences. However, the molecular method utilizing partial *cpn60* sequences is a sensitive and specific technique that remains time-consuming. The MALDI Biotyper, based on a sensitive, fast and widely-used MALDI-TOF MS method, demonstrated capability to reliably differentiate the phylogenetically diverse groups composed of species *G. leopoldii*/*G. swidsinskii* and *G. vaginalis*/*G. piotii*. Our results confirmed recent findings that sialidase NanH3 is responsible for sialidase activity in a collection of 34 *Gardnerella* isolates. *G. leopoldii* and *G. swidskinskii* species do not contain any genes coding for sialidases and display a sialidase activity-negative phenotype. The β-galactosidase activity was detected only in *G. vaginalis* strains.

## Data Availability

Data are contained within the article and supplementary material.

## Supplementary Materials

The following are available online at https://www.mdpi.com/2076-0817/10/3/277/s1, Table S1: Pairwise distances between *cpn60* UT nucleotide sequences, Table S2: Comparison of MALDI-TOF spectra of *Gardnerella* isolates and calculation of log(score), Table S3: Characteristics of *Gardnerella* isolates, Table S4: *Gardnerella* species detected in the characterized vaginal samples, Figure S1: PCR detection of the *nanH2* and *nanH3* genes.
