# Supplementary material for: Discrimination of Gardnerella Species by Combining MALDI-TOF Protein Profile, Chaperonin cpn60 Sequences, and Phenotypic Characteristics
Source: Pathogens. 2021 Mar 1;10(3):277. doi: 10.3390/pathogens10030277 (PMC7998583; doi:10.3390/pathogens10030277)
Supplement: Supplementary file 1 [file pathogens-10-00277-s001.zip › Table S4.pdf]

Table S4. *Gardnerella* species detected in the characterized vaginal samples

Vaginal sample[a]	Nugent score[b]	Subgroups/clades in vaginal sample[c]	Isolate no.[d]	Clade[e]	*Gardnerella* species	
					MALDI Biotyper	*cpn60* UT
046S1	7	1+2+4	46.6	1	*G.vaginalis*	*G.vaginalis*
047S1	10	1+4	47.3	1	*G.vaginalis*	*G.vaginalis*
056S1	6	1+4	56.1	1	*G.vaginalis*	*G.vaginalis*
057S1	10	1	57.1	1	*G.vaginalis*	*G.vaginalis*
058S1	9	1+2+4	58.1	4	*G.leopoldii/G.swidsinskii*	*G.leopoldii*
			58.4	1	*G.vaginalis*	*G.vaginalis*
			58.7	2	*G.piotii*	*G.piotii*
058S2	5	4	58.2.1	4	*G.leopoldii/G.swidsinskii*	*G.leopoldii*
			58.2.3	1	*G.vaginalis*	*G.vaginalis*
060S1	10	2+4	60.1	2	*G.piotii*	*G.piotii*
063S1	8	1+2+4	63.1	4	*G.leopoldii/G.swidsinskii*	*G.leopoldii*
			63.2	2	*G.piotii*	*genome sp.3*
065S1	9	1+2+4	65.2	2	*G.piotii*	*genome sp.3*
076S1	6	1+2+4	76.2	1	*G.vaginalis*	*G.vaginalis*
078S1	5	2+4	78.1	2	*G.piotii*	*G.piotii*
079S1	4	1	79.2	1	*G.vaginalis*	*G.vaginalis*
082S1	9	1+2+4	82.1	4	*G.leopoldii/G.swidsinskii*	*G.leopoldii*
			82.2	2	*G.piotii*	*genome sp.3*
083S1	6	1+2	83.1	1	*G.vaginalis*	*G.vaginalis*
084S1	9	1+2+4	84.1	1	*G.vaginalis*	*G.vaginalis*
			84.4	2	*G.piotii*	*G.piotii*
			84.5	1	*G.vaginalis*	*G.vaginalis*
			84.6	2	*G.piotii*	*G.piotii*
086S1	6	1+2+3+4	86.1	ND	*G.vaginalis*	*G.vaginalis*
			86.3	2	*G.piotii*	*genome sp.3*
			86.5	2	*G.piotii*	*G.piotii*
088S1	10	1+2+3+4	88.2	4	*G.leopoldii/G.swidsinskii*	*G.leopoldii*
099S1	3	1+2+3+4	99.1	4	*G.leopoldii/G.swidsinskii*	*G.leopoldii*
105S1	3	1+4	105.1	1	*G.vaginalis*	*G.vaginalis*
106S1	9	1+2+3+4	106.3	4	*G.leopoldii/G.swidsinskii*	*G.swidsinskii*
			106.5	1	*G.vaginalis*	*G.vaginalis*
107S1	10	1+2+4	107.1	4	*G.leopoldii/G.swidsinskii*	*G.swidsinskii*
114S1	10	1+2+4	114.2	1	*G.vaginalis*	*G.vaginalis*

[a] Collection of vaginal samples is described in [14].

[b] Vaginal samples were subjected to Gram-staining and microscopy to assess their Nugent score as described in [14].

[c] DNA extracted from the characterized vaginal samples was used for subtyping of clades by PCR as described in [10,14].

[d] Number of isolated *Gardnerella* strain from the vaginal samples [14].

[e] Isolates from individual colonies were subtyped by clade specific PCR [10,14].
