# Supplementary material for: Discrimination of Gardnerella Species by Combining MALDI-TOF Protein Profile, Chaperonin cpn60 Sequences, and Phenotypic Characteristics
Source: Pathogens. 2021 Mar 1;10(3):277. doi: 10.3390/pathogens10030277 (PMC7998583; doi:10.3390/pathogens10030277)
Supplement: Supplementary file 1 [file pathogens-10-00277-s001.zip › Table S3.pdf]

Table S3. Characteristics of *Gardnerella* isolates

Isolate	Clade	Vaginolysin (VLY)		β-galactosidase activity	Biofilm former[b]	Sialidase activity assays		Sialidase genes			*Gardnerella* species	
		Gene	VLY production[a]			Filter spot	Fluorometric[c]	*nanH1*	*nanH2*	*nanH3*	MALDI Biotyper	*cpn60* UT
46.6	1	+	132.7± 20.9	positive	weak	–	0	+	–	–	*G.vaginalis*	*G.vaginalis*
47.3	1	+	125.6 ± 7.9	positive	weak	+	9450±305	+	–	+	*G.vaginalis*	*G.vaginalis*
56.1	1	+	270.3 ± 6.6	positive	strong	–	0	+	–	–	*G.vaginalis*	*G.vaginalis*
57.1	1	+	381.2 ± 36.6	positive	weak	–	30±6	+	–	–	*G.vaginalis*	*G.vaginalis*
58.2.3	1	+	2062.5 ± 147.9	positive	moderate	–	0	+	–	+	*G.vaginalis*	*G.vaginalis*
58.4	1	+	1900.8 ± 89.7	positive	moderate	+	1628±70	+	–	+	*G.vaginalis*	*G.vaginalis*
76.2	1	+	61.9 ± 2.9	positive	strong	–	13±2	+	–	–	*G.vaginalis*	*G.vaginalis*
79.2	1	+	20.7 ± 4.5	positive	moderate	–	0	+	–	–	*G.vaginalis*	*G.vaginalis*
83.1	1	+	126.8 ± 25.4	positive	weak	–	5±1	+	–	–	*G.vaginalis*	*G.vaginalis*
84.1	1	+	105.0 ± 24.9	positive	weak	–	4±1	+	–	–	*G.vaginalis*	*G.vaginalis*
84.5	1	+	67.4 ± 9.8	positive	moderate	–	24±4	+	–	–	*G.vaginalis*	*G.vaginalis*
105.1	1	+	68.0 ± 5.3	positive	weak	–	0	+	–	–	*G.vaginalis*	*G.vaginalis*
106.5	1	+	213.6 ± 21.5	positive	strong	+	121±7	+	–	+	*G.vaginalis*	*G.vaginalis*
114.2	1	+	192.2 ± 25.2	positive	strong	–	36±2	+	–	–	*G.vaginalis*	*G.vaginalis*
86.1	–	–	0	positive	moderate	–	0	+	–	–	*G.vaginalis*	*G.vaginalis*
58.7	2	–	0	negative	non	+	525±28	+	+	+	*G.piotii*	*G.piotii*
60.1	2	+	101.5 ± 5.1	negative	strong	–	34±3	+	–	+	*G.piotii*	*G.piotii*
63.2	2	+	1809.8 ± 161.3	negative	strong	+	362±158	+	–	+	*G.piotii*	*genome sp.3*
65.2	2	+	1151.6 ± 12.7	negative	non	+	867±54	+	–	+	*G.piotii*	*genome sp.3*
78.1	2	+	141.0 ± 32.6	negative	weak	+	250±68	+	–	+	*G.piotii*	*G.piotii*
82.2	2	+	56.6 ± 4.7	negative	strong	+	43±18	+	–	+	*G.piotii*	*genome sp.3*
84.4	2	–	0	negative	weak	+	455±126	+	+	+	*G.piotii*	*G.piotii*
84.6	2	–	0	negative	weak	+	317±40	+	+	+	*G.piotii*	*G.piotii*
86.3	2	+	743.8 ± 49.2	negative	strong	+	727±41	+	–	+	*G.piotii*	*genome sp.3*
86.5	2	–	0	negative	weak	+	550±24	+	+	–	*G.piotii*	*G.piotii*
58.1	4	+	3.71 ± 0.33	negative	non	–	0	–	–	–	*G.leopoldii/G.swidsinskii*	*G.leopoldii*
58.2.1	4	+	2.0 ± 0.2	negative	non	–	0	–	–	–	*G.leopoldii/G.swidsinskii*	*G.leopoldii*
63.1	4	+	0	negative	non	–	0	–	–	–	*G.leopoldii/G.swidsinskii*	*G.leopoldii*

82.1	4	+	7.6 ± 0.5	negative	non	–	0	–	–	–	*G.leopoldii/G.swidsinskii*	*G.leopoldii*
88.2	4	+	0	negative	weak	–	0	–	–	–	*G.leopoldii/G.swidsinskii*	*G.leopoldii*
99.1	4	+	44.1 ± 12.3	negative	weak	–	0	–	–	–	*G.leopoldii/G.swidsinskii*	*G.leopoldii*
106.3	4	+	797.8 ± 90.9	negative	moderate	–	0	–	–	–	*G.leopoldii/G.swidsinskii*	*G.swidsinskii*
107.1	4	+	298.8 ± 28.5	negative	weak	–	0	–	–	–	*G.leopoldii/G.swidsinskii*	*G.swidsinskii*
GV37	4	+	>800	negative	ND	–	0	–	–	–	*G.leopoldii/G.swidsinskii*	*G.swidsinskii*

[a] Values are VLY concentration (ng/mL) normalized to OD_{600} of the cultures expressed as the mean of three biological replicates and two technical replicates (n = 6) per each isolate ± standard deviation [14].

[b] Isolates were classified based on biofilm-forming ability, as described in [14].

[c] Values are sialidase activity normalized to OD_{600} of the cultures expressed as the mean of three biological and two technical replicates (n = 6) per each isolate ± standard deviation. Negative values adjusted to 0 [14].

ND, not detected
