# Supplementary figures and images for: Discrimination of Gardnerella Species by Combining MALDI-TOF Protein Profile, Chaperonin cpn60 Sequences, and Phenotypic Characteristics
Source: Pathogens. 2021 Mar 1;10(3):277. doi: 10.3390/pathogens10030277 (PMC7998583; doi:10.3390/pathogens10030277)

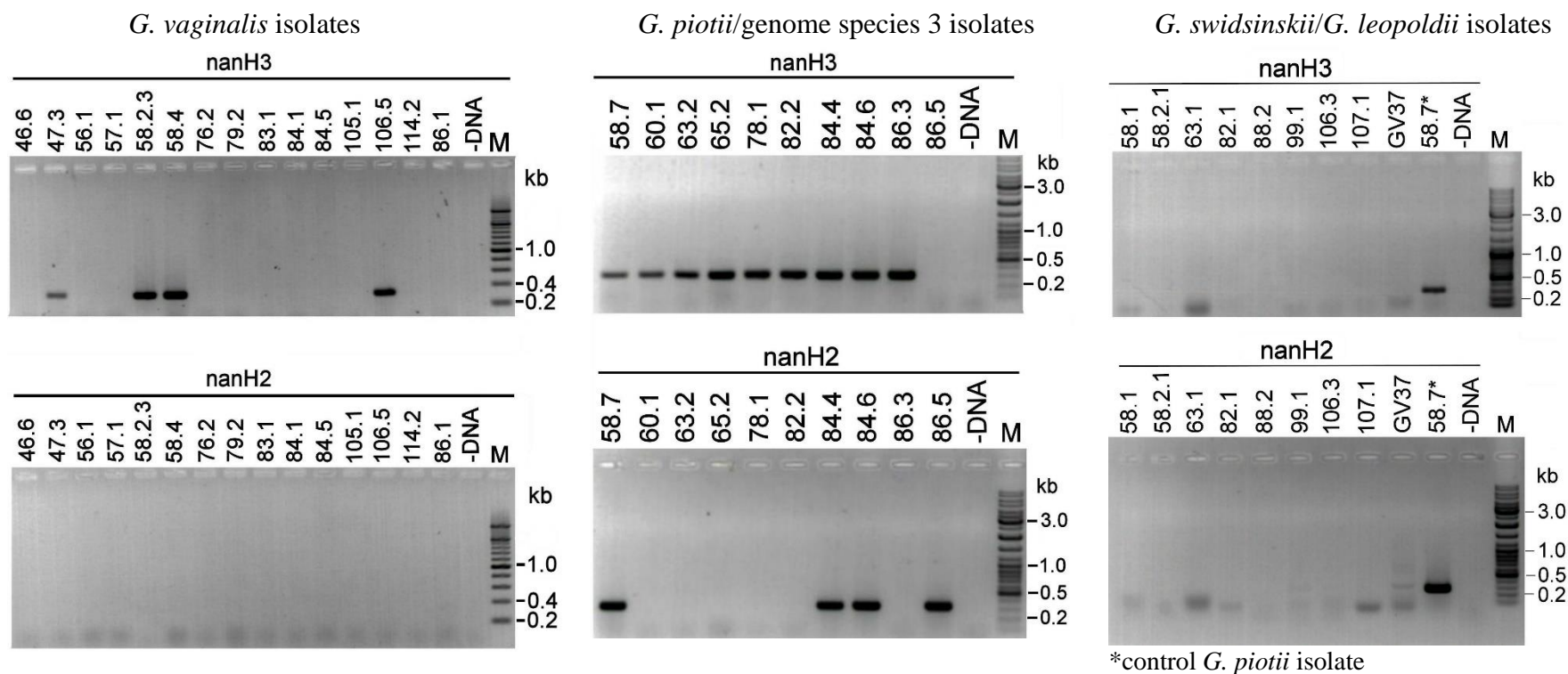

**Figure S1.** PCR detection of the *nanH2* and *nanH3* genes

Supplement: Supplementary file 1 [file pathogens-10-00277-s001.zip › Figure S1.pdf]
